# Evaluation of patient satisfaction with the day surgical services in an Irish teaching hospital

**DOI:** 10.1186/1753-6561-6-S4-O5

**Published:** 2012-07-09

**Authors:** YH Soon, G Gethin, B Meshkat, TN Walsh, S Cowman, M Wiley, A Brick, E Clarke

**Affiliations:** 1 Royal College of Surgeons in Ireland

## Introduction

Advancements in surgical techniques and anaesthetic agents in recent decades have allowed for the rapid expansion of day surgical services internationally. The aim of this study is to evaluate the patient's satisfaction (PS) with the day surgical services in an Irish teaching hospital.

## Methods

The study design was a survey, utilizing a phone questionnaire. 70 consecutively presenting patients undergoing 15 day surgery procedures in an Irish teaching hospital were invited to participate. Data collection was completed between June and July 2011. The survey evaluated 57 aspects of the DS process. Patients were asked to rate their level of satisfaction using a 5-point LIKERT scale ranging from 1-very dissatisfied to 5-very satisfied.

## Results

70 patients consented to study participation, of which 85.57% (n=60) responded when phoned. Of these; 40% (n=24) male, 60% (n=36) female, mean age 47 years (range 17-84 years), 50% (n=30) had local anaesthetic procedures (LA) and 50% (n=30) general anaesthetic procedures (GA). Pre-operatively, patients were least satisfied with the waiting time: 35.59% (n=21) very satisfied; while 66.66% (n=40) were very satisfied with the nursing staff. 81.66% (n=49) of the patients were very satisfied with the medical care provided. Post-operatively, 94.44% (n=51) were very satisfied with the follow-up arrangements. However, only 54.55% (n=24) were very satisfied with the time taken to obtain results for x-rays and other tests. Regarding the supporting structures to the day surgery unit (DSU), patients were most satisfied with the cleanliness of the unit, where 86.66% (n=52) were very satisfied, while only 39.13% (n=18) were very satisfied with parking arrangements. Additionally, 84.21% (n=57) were very satisfied with the information leaflet regarding the procedure.

## Conclusions

Overall, patients were very satisfied with the care provided and service provision in the DSU. As day surgery services are expanded into the future, this study provides valuable insights into the current areas for improvement and development. Although the methodology used was very labour-intensive, the response rate was very high. Higher recruitment through e-mail or text messaging could have proven to be more time saving and cost efficient. However this might be at the expense of lower response rates.

